# Oleic acid induces down-regulation of the granulosa cell identity marker FOXL2, and up-regulation of the Sertoli cell marker SOX9 in bovine granulosa cells

**DOI:** 10.1186/s12958-017-0276-z

**Published:** 2017-07-26

**Authors:** Vengala Rao Yenuganti, Jens Vanselow

**Affiliations:** Institute of Reproductive Biology, Leibniz Institute for Farm Animal Biology (FBN), Wilhelm-Stahl-Allee 2, 18196 Dummerstorf, Germany

**Keywords:** Negative energy balance, *PTGS2*, *ESR2*, Cell identity

## Abstract

During negative energy balance, the concentration of different fatty acids, especially of oleic acid (OA) increases in the follicular fluid of cattle. Previously, we showed that OA induced morphological, physiological and molecular changes in cultured bovine granulosa cells. In our present study we analyzed effects of OA on the expression of markers for granulosa and Sertoli cell identity, FOXL2 and SOX9, respectively, in addition to effects on the FOXL2 regulated genes ESR2, FST, PTGS2 and PPARG. The results showed that OA down-regulated FOXL2, ESR2, FST and PPARG but up-regulated PTGS2 and SOX9. From these data we conclude that OA can compromise granulosa cell functionality and may initiate trans-differentiation processes in bovine granulosa cells. This novel mechanism may be causally involved in postpartum fertility problems of lactating dairy cows.

## Introduction

High-yielding dairy cows suffer from negative energy balance after parturition [1, 2]. To meet their energy requirements in spite of low blood glucose levels lactating dairy cows mobilize fat from adipose tissue. Consequently, levels of free fatty acids like palmitic acid (PA, 16:0), stearic acid (SA, 18:0), oleic acid (OA, 18:1) and of β-hydroxybutyric acid increase in the plasma and follicular fluid [3, 4], which can cause decreased milk yield and increased vulnerability to infections, metabolic diseases and sub-fertility. Short-term fasting of cattle also increases the levels of different fatty acids in the follicular fluid and especially of OA [5]. In our previous study, we showed that OA at physiological concentrations remarkably affected the cell morphology, reduced the abundance of functionally important transcripts as *FSHR, LHCGR, STAR, CYP11A1, HSD3B1* and *CYP19A,* and decreased the production of 17-beta estradiol (E2) in cultured bovine granulosa cells (GC). In contrast, the expression of *CD36* and *SLC27A1*, encoding fatty-acid transporters was increased [6]. Sahmi et al. [7] showed that the transcription factor and GC identity marker *FOXL2* is vital for higher activity of the *CYP19A1* promoter P2 in bovine GC. So we hypothesized that OA may affect the expression of *FOXL2* and thus the GC identity. *FOXL2* is involved in the regulation of the Sertoli cell marker *SOX9* in the ovary at early developmental and adult stages [8–12]. In adult mice the deletion of *FOXL2* in GC increases the expression of *SOX9* and development of seminiferous tubule-like structures in the ovary [11]. Also the deletion of estrogen receptors or low levels of estrogen resulted in a similar phenotype of GC [12–14], thus demonstrating that the expression of *SOX9* in ovaries is normally suppressed via *ESR2* (estrogen receptor 2) and E2 along with *FOXL2*. *FOXL2* also positively regulates the expression of *ESR2*, *PPARG* and *FST*, whereas it negatively regulates the expression of *PTGS2* [12, 15, 16]. These genes are essentially involved in normal GC function [17–21]. *SOX9* is a very early and permanently expressed marker of Sertoli cells [22]. Loss- and gain-of-function studies revealed that *SOX9* is required for Sertoli cell differentiation. Deletion of *SOX9* in XY gonads leads to male-to-female sex reversal and misexpression in XX mice to female-to-male sex reversal [23, 24], thus demonstrating that *SOX9* plays an essential role during testis differentiation processes [25–28]. On the other hand, the loss of the SRY-dependent SOX9 inducibility in bipotential gonadal cells is the earliest sign of pre-granulosa cell differentiation in XX gonads [29].

During the present study we analysed effects of OA in cultured bovine GC on *FOXL2* and *SOX9*, which are essentially involved in the maintenance of granulosa and Sertoli cell identity, respectively. In addition, we also studied effects of OA on other functionally important genes that are regulated by *FOXL2* like *ESR2*, *PPARG, FST* and *PTGS2*.

## Materials and methods

### Serum free bovine granulosa cell culture

As an experimental model GC were aspirated from small to medium sized follicles (2–6 mm) from slaughterhouse material, plated on collagen coated 24-well plates with 1.25 × 10^5^ viable cells and cultured in serum-free α-MEM containing L-Glutamin (2 mM), sodium bicarbonate (0.084%), BSA (0.1%), HEPES (20 mM), sodium selenite (4 ng/ml), transferrin (5 μg/ml), insulin (10 ng/ml), non-essential amino acids (1 mM), penicillin (100 IU) and streptomycin (0.1 mg/ml; Biochrom, Berlin, Germany) with FSH and IGF1 stimulation and androstenedione (2 μM) supplementation (Sigma Aldrich, Steinheim, Germany) at 37 °C in a 5% CO_2_ atmosphere according to our previous studies [6, 30, 31]. Media were replaced with fresh media including all respective supplements every other day. OA was added from the first change of media (i.e. after 2 days in culture) to the end of culture after 8 days. The amount of added OA (dissolved in ethanol as described previously [6]) in all the experiments was 400 μM, which is in the range of physiological concentrations after fasting [5] and reproducibly affected hormone production and gene expression of cultured GC [6]. As a vehicle control, cells were treated with equal volumes of ethanol corresponding to OA treatment.

### RNA isolation, cDNA synthesis and real- time PCR

RNA was isolated with the Nucleo Spin® RNA II Kit according to the manufacturer’s instructions and quantified with a NanoDrop1000 Spectrophotometer (Thermo Scientific, Bonn, Germany). The cDNA was prepared by using the SensiFAST cDNA Synthesis Kit (Bioline, Luckenwalde, Germany) from 200 ng RNA as per the manufacture’s protocol. Transcript abundance of *FOXL2, SOX9, ESR2, FST, PPARG* and *PTGS2* was analyzed as described in our previous studies [6, 31] by real time PCR with SensiFast SYBR No-ROX (Bioline) and gene-specific primers (listed in Table 1) in a Light Cycler 96 instrument (Roche,Mannheim, Germany). The abundance of all transcripts was calculated relative to transcripts of the TATA-binding protein (TBP) as an appropriate housekeeping gene [32].

**Table 1:** List of primers used for transcript quantification by qPCR

Name	Sequence	Size (bp)	NCBI accession no.
*ESR2* Forward	GGTCAATCCATCCTACCCCT	264	NM_174051.3
*ESR2* Reverse	TTCACGCCAAGGACTCTTTT		
*FOXL2* Forward	AGCCAAGTTCCCGTTCTACG	140	NM_001031750.1
*FOXL2* Reverse	GGTCCAGCGTCCAGTAGTTG		
*FST* Forward	GCACTGGCCGCCTGAGCACCT	191	NM_175801
*FST* Reverse	TGGGGCACAGACGCAGCGGG		
*PPARG* forward	TATCCCCGGCTTTGTGAACC	288	NM_181024.2
*PPARG* Reverse	GGGCGGTCTCCACTGAGAAT		
*PTGS2* Forward	TACAGCACTTGAGTGGCTATCAC	317	NM_174445
*PTGS2* Reverse	CTGGTCAATTGAAGCCTTTGATAC		
*SOX9* Forward	ACCTGGAACTTCAGTGGCG	147	XM_010816647.1
*SOX9* Reverse	CCAAGTAGGGGAAGGCGAAT		
*TBP* Forward	GCCTTGTGCTTACCCACCAACAGTTC	200	NM_001075742.1
*TBP* Reverse	TGTCTTCCTGAAACCCTTCAGAATAGGG		

### Isolation of soluble cell extracts and immunoblotting

Isolation of soluble cell extracts and immunoblotting for detection of FOXL2 and SOX9 proteins, and of GAPDH as a loading control, was done as described previously [31]. Primary antibodies were from Thermo Fisher Scientific, Dreieich, Germany (FOXL2, Cat# PA1-31950) or Cell Signaling Technology, Frankfurt/main, Germany (SOX9, Cat# CST 7074, GAPDH, Cat# CST 3683), secondary antibodies from Cell Signaling Technology (anti-rabbit IgG, HRP-linked Antibody, Cat# CST) or Santa Cruz, Heidelberg, Germany (donkey anti-goat IgG-HRP, Cat# sc-2020).

### Statistical analysis

Data of all experiments were analysed by paired t-test using the GraphPad prism 5.0 software. All experiments were conducted three times independently.

## Results

**Fig. 1:**
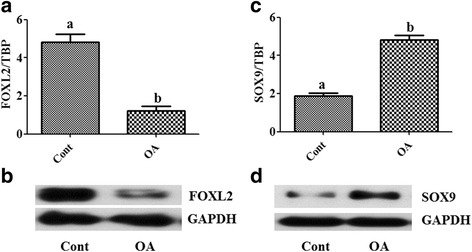
Effects of oleic acid on the expression of the granulosa cell marker FOXL2 and the Sertoli cell marker SOX9 in cultured granulosa cells. Oleic acid (OA) induced down-regulation of *FOXL2* mRNA and protein abundance (**a** and **b**), respectively, and up-regulation of *SOX9* mRNA and protein, respectively (**c** and **d**). GAPDH protein is shown as a loading control. Different letters indicate significant differences between treatment groups (mean transcript abundance relative to TBP ± standard error; different letters indicate significantly different means if *P* < 0.05, unpaired t-test from three independent experiments). Cont denotes vehicle control and OA denotes oleic acid treatment

**Fig. 2:**
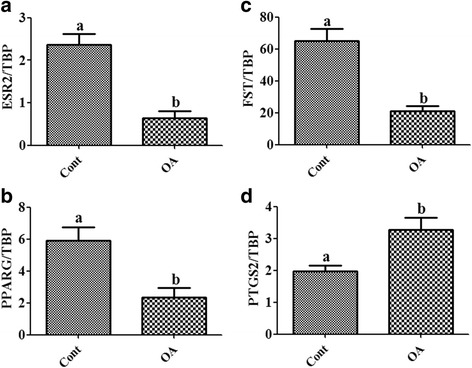
Effect of oleic acid on the expression of FOXL2 regulated genes. (**a**, **b**, **c** and **d**) OA induced regulation of *ESR2, PPARG, FST* and *PTGS2* transcript abundance levels. Different letters indicate significant differences between treatment groups (mean transcript abundance relative to TBP ± standard error; different letters indicate significantly different means if *P* < 0.05, unpaired t-test from three independent experiments). Cont denotes vehicle control and OA denotes oleic acid treatment

The results showed that OA clearly down regulated the transcript abundance and protein levels of *FOXL2*, but up regulated those of *SOX9* (Fig. 1). As OA inhibited *FOXL2* mRNA and protein levels, we analyzed the effects of OA on the expression of genes that are involved in different functions of GC and are regulated by *FOXL2* as *FST, ESR2, PTGS2* and *PPARG* [12]. The results showed that OA significantly down regulated the expression of *ESR2, PPARG* and *FST* as compared with vehicle treated controls, whereas *PTGS2* was increased (Fig. 2).

## Discussion

In the present study OA markedly reduced the levels of the transcription factors *FOXL2* and *ESR2*, and increased the expression of *SOX9*. It is a very well established fact that *FOXL2* is involved in maintaining the identity of GC [12]. The deletion of *FOXL2* in GC of adult mice resulted in the development of seminiferous tubule-like structures along with the expression of Sertoli-cell marker genes in the ovary [11]. Georges et al. [12] showed that knockdown of *FOXL2* or *ESR2* leads to a rise of *SOX9* levels and in case of a knockdown of both genes, *SOX9* levels became even more effectively up-regulated. The same study also showed that knockdown of *ESR1* has no clear effect on *SOX9* repression following E2 treatment, but *ESR2* knockdown resulted in an increase of *SOX9* expression, whereas *SOX9* expression was markedly increased in the absence of *FOXL2*. Thus, *FOXL2* and *ESR2*/E2 repress *SOX9* expression in follicular GC independent of each other [12]. In addition to this, it has been shown that *FOXL2* regulates many genes like *FST*, *PTGES2* and *PPARG* [12]*.* Our results showed that OA down regulated the expression of *FST* and *PPARG* along with that of *FOXL2.* The expression of *FST* in the ovary and GCs is regulated by *FOXL2* [8, 16]. Knockdown of *FOXL2* leads to decreased expression of *PTGS2* and *PPARG* in murine primary GC [12]. Our results showed that OA induced down-regulation of *PPARG* along with *FOXL2* but not of *PTGS2*, which was increased by OA treatment. Kim et al. [15] showed that *FOXL2* represses the expression of *PTGS2,* which would be in line with our data. This suggests that OA may up-regulate *SOX9* expression by reducing the levels of *FOXL2*, *ESR2* and of E2. The data of our present study demonstrate that the expression of *FOXL2, ESR2, PPARG,* and *FST* was clearly decreased. In contrast, the expression of *PTGS2* and in particular of *SOX9* was increased by OA. Accordingly, we propose that OA induces the Sertoli cell marker SOX9 in GC eventually leading to a partial loss of GC identity, which may severely compromise GC’s functionality.

## Conclusions

Our data suggest that (i) OA treatment reduced the expression of *FOXL2*. (ii) The inhibition of *FOXL2* expression in turn may lead to the down-regulation of *ESR2, FST* and *PPARG*. (iii) The low levels of *FOXL2, ESR2* and of E2 may overcome transcriptional suppression of the Sertoli cell marker *SOX9* and thus induce trans-differentiation-like processes, which would severely compromise GC functionality. This novel observation could partly explain the negative effects of increased OA concentrations on post-partum fertility in dairy cows.

## Abbreviations

CYP19A1: Cytochrome P450, family 19, subfamily A, polypeptide 1
E2: 17-beta estradiol
ESR2: Estrogen receptor 2
FOXL2: Forkhead box protein L2
FSH: Follicle-stimulating hormone
FST: Follistatin
GAPDH: Glyceraldehyde-3-phosphate dehydrogenase
GC: Granulosa cells
IGF-1: Insulin-like growth factor 1
PPARG: Peroxisome proliferator-activated receptor gamma
PTGES2: Prostaglandin E synthase 2
SOX9: SRY (sex determining region Y)-box 9
TBP: TATA-binding protein
